# Fermented Fish Collagen Attenuates Melanogenesis via Decreasing UV-Induced Oxidative Stress

**DOI:** 10.3390/md22090421

**Published:** 2024-09-15

**Authors:** Kyung-A Byun, So Young Lee, Seyeon Oh, Sosorburam Batsukh, Jong-Won Jang, Bae-Jin Lee, Kyoung-min Rheu, Sichao Li, Min-Seok Jeong, Kuk Hui Son, Kyunghee Byun

**Affiliations:** 1 Department of Anatomy & Cell Biology, College of Medicine, Gachon University, Incheon 21936, Republic of Korea; 2 LIBON Inc., Incheon 22006, Republic of Korea; 3 Functional Cellular Networks Laboratory, Lee Gil Ya Cancer and Diabetes Institute, Gachon University, Incheon 21999, Republic of Korea; 4 Department of Thoracic and Cardiovascular Surgery, Gachon University Gil Medical Center, Gachon University, Incheon 21565, Republic of Korea; 5 Department of Health Sciences and Technology, Gachon Advanced Institute for Health & Sciences and Technology (GAIHST), Gachon University, Incheon 21999, Republic of Korea; 6 Marine Bioprocess Co., Ltd., Busan 46048, Republic of Korea

**Keywords:** fermented fish collagen, glycine, UV-induced model

## Abstract

Excessive melanogenesis leads to hyperpigmentation-related cosmetic problems. UV exposure increases oxidative stress, which promotes melanogenesis-related signal pathways such as the PKA, microphthalmia-associated transcription factor (MITF), tyrosinase (TYR), tyrosinase-related protein-1 (TRP1), and tyrosinase-related protein-2 (TRP2) pathways. Glycine is a source of endogenous antioxidants, including glutathione. Fermented fish collagen (FC) contains glycine; thus, we evaluated the effect of FC on decreasing melanogenesis via decreasing oxidative stress. The glycine receptor (GlyR) and glycine transporter-1 (GlyT1) levels were decreased in UV-irradiated keratinocytes; however, the expression levels of these proteins increased upon treatment with FC. The FC decreased oxidative stress, as indicated by the decreasing expression of NOX1/2/4, increased expression of GSH/GSSG, increased SOD activity, and decreased 8-OHdG expression in UV-irradiated keratinocytes. Administration of conditioned media from FC-treated keratinocytes to melanocytes led to decreased p38, PKC, MITF, TRP1, and TRP2 expression. These changes induced by the FC were also observed in UV-irradiated animal skin. FC treatment increased the expression of GlyR and GlyT, which was accompanied by decreased oxidative stress in the UV-irradiated skin. Moreover, the FC negatively regulated the melanogenesis signaling pathways, leading to decreased melanin content in the UV-irradiated skin. In conclusion, FC decreased UV-induced oxidative stress and melanogenesis in melanocytes and animal skin. FC could be used in the treatment of UV-induced hyperpigmentation problems.

## 1. Introduction

Skin is the first defense organ against various environmental factors such as ultraviolet (UV) light; however, excessive exposure to UV increases the generation of reactive oxygen species (ROS) in the skin [1,2,3,4]. Excessive ROS are removed by various antioxidant enzymes such as superoxide dismutase (SOD), catalase, and glutathione peroxidase [5,6,7]. When the balance between the generation and removal of ROS shifts toward increased generation, oxidative stress increases, which in turn stimulates melanogenesis [8].

Melanogenesis is the process of synthesis of melanin in melanocytes [9,10,11,12]. UV initiates melanogenesis by stimulating keratinocytes to secrete α-melanocyte-stimulating hormone (αMSH), which eventually binds to the melanocortin 1 receptor (MC1R) on melanocytes [13]. Activated MC1R upregulates PKA, microphthalmia-associated transcription factor (MITF), tyrosinase (TYR), tyrosinase-related protein-1 (TRP1), and tyrosinase-related protein-2 (TRP2) expressions, which are key factors of melanogenesis [9,10,11,12,14].

UV also increases ROS generation via the upregulation of nicotinamide adenine dinucleotide phosphate (NADPH) oxidase (NOX) expression [15,16,17]. ROS in keratinocytes may be transferred to the melanocytes [18].

Increased oxidative stress also leads to upregulation of MITF and TYR expression, which increases melanogenesis [19,20,21]. ROS also increases MAPK expression, including p38 expression, which activates MITF [8]. Although the proper generation of melanin is necessary to protect the skin from UV, excessive melanogenesis causes various skin problems, such as melasma and post-inflammatory hyperpigmentation [22,23].

Supplements of collagen, such as hydrolyzed collagen, have been used to promote skin health [24]. After ingestion of hydrolyzed collagen, it degrades into dipeptides or tripeptides such as proline-hydroxyproline (Pro-Hyp), glycine-proline (Gly-Pro), or Gly-Pro-Hyp [25,26,27].

Glycine is an antioxidant and exhibits cytoprotective effects in various organs [28,29,30,31,32]. The cytoprotective effect of glycine is associated with increasing activity of the glycine receptor (GlyR) and glycine transporter-1 (GlyT1) [32,33,34]. GlyT has a high affinity for glycine and transports glycine into the cytosol [33]. Glycine is needed to generate glutathione, which is one of the main endogenous antioxidants [35].

Indeed, it has been reported that glycine supplements activate GlyT, which transports glycine into the cytosol and increases the synthesis of glutathione [36]. In addition, glycine activates GlyR, which eventually inhibits NOX and decreases oxidative stress [36]. By decreasing oxidative stress, glycine thus exhibits a protective effect against high-glucose-induced cell injury in β-cells [36].

Fermented fish collagen (FC) is generated by fermentation of hydrolyzed fish collagen with *Lactobacillus plantarum* BJ21 and *Lactobacillus brevis* BJ20. When we evaluated FC components, glycine was found to be one of main components of FC. Thus, we hypothesized that FC could upregulate GlyR and GlyT expression, which would decrease NOX activity and oxidative stress in UV-irradiated skin. Decreased oxidative stress would in turn lead to decreased p38 and PKC expression, which would eventually cause a decrease in the MITF/TYR/TRP1/TRP2 pathways—which are the main pathways of melanogenesis. We evaluated this hypothesis using UV-irradiated keratinocyte and melanocyte models and UV-irradiated animal models.

## 2. Results

### 2.1. FC Increased the Expressions of GlyR and GlyT in the UV-Irradiated Keratinocyte

First, FC concentration was determined based on cell viability and effectiveness in increasing SOD activity. The cytotoxic effects of varying concentrations of FC (1–100,000 μg/mL) on keratinocytes (HaCaT) were assessed after 48 h of incubation (Figure S1A). No cytotoxicity was observed at concentrations below 100,000 μg/mL of FC (Figure S1B).

The SOD activity was evaluated in UV-irradiated keratinocytes (Figure S1C). The SOD activity was significantly decreased upon UV irradiation in keratinocytes. The SOD activity was increased after FC treatment. The increase in SOD activity upon FC treatment was not significantly different among cells treated with 250, 500, and 1000 μg/mL of FC (Figure S1D).

**Figure 1:**
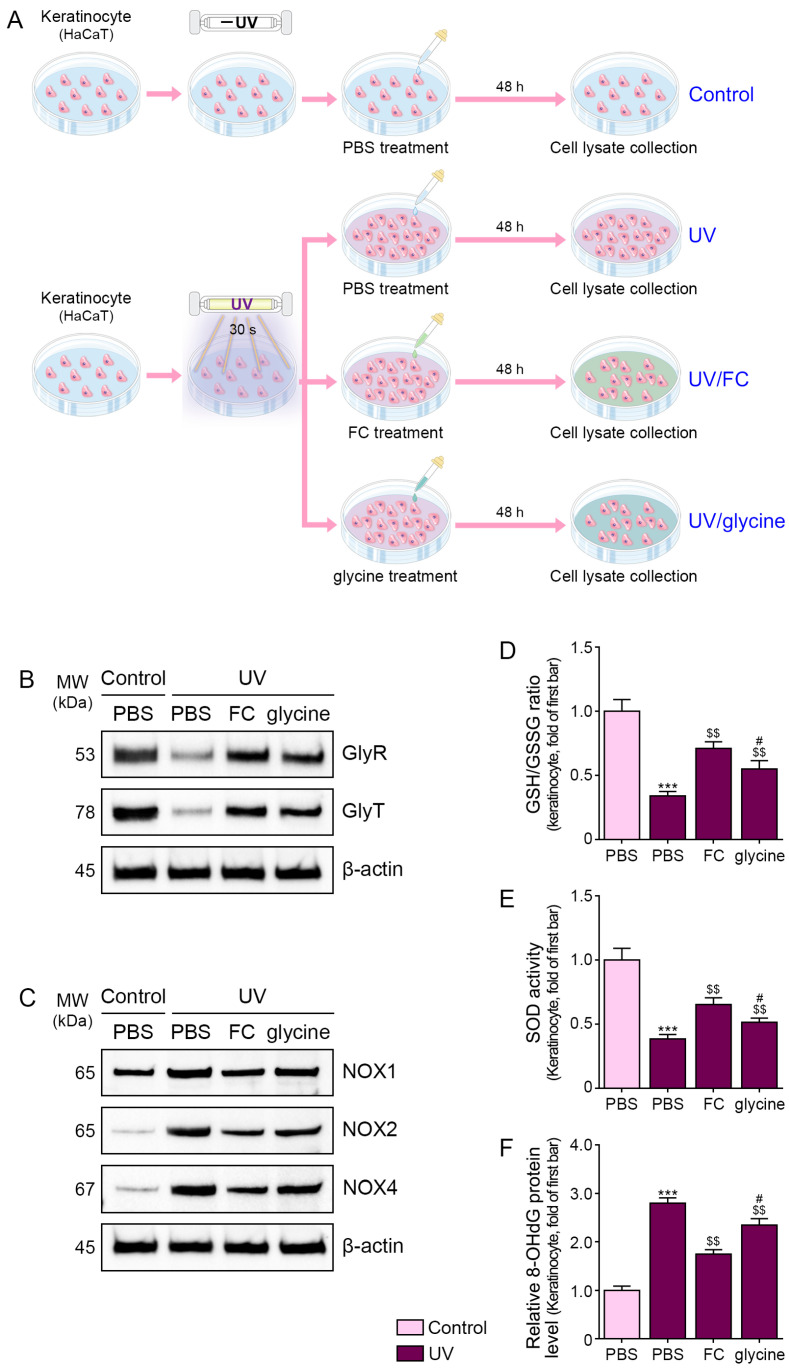
Regulation of GlyR and GlyT expression and oxidative stress by FC and glycine in UV-exposed keratinocytes. (**A**) Schematic diagram of the treatment of UV-exposed keratinocytes with FC and glycine. (**B**) Protein expression of GlyR and GlyT in UV-exposed keratinocytes following FC and glycine treatment. (**C**) Protein expression of NOX1/2/4 in UV-exposed keratinocytes following FC and glycine treatment. (**D**) The increased level of GSH/GSSG ratio in UV-exposed keratinocytes following FC and glycine treatment. (**E**) The SOD activity in UV-exposed keratinocytes following FC and glycine treatment. (**F**) The increased level of 8-OHdG in UV-exposed keratinocytes following FC and glycine treatment. Data are presented as the mean ± SD of three independent experiments. ***, *p* < 0.001, first bar vs. second bar; $$, *p* < 0.01, vs. second bar; #, *p* < 0.05, vs. third bar (Mann–Whitney U test). FC, fermented fish collagen; GlyR, glycine receptor; GlyT, glycine transporter; GSH, glutathione; GSSG, oxidized glutathione; NOX, nicotinamide adenine dinucleotide phosphate oxidase; PBS, phosphate-buffered saline; SD, standard deviation; SOD, superoxide dismutase; UV, ultraviolet; 8-OHdG, 8-hydroxy-2-deoxyguanosine.

Consequently, 250 μg/mL of FC was identified as a suitable concentration for subsequent experiments. We also compared the effect of FC treatment with that of a single treatment of glycine. As 250 μg/mL of FC contains 2.5 μg/mL of glycine, we used this concentration of glycine (Figure 1A).

The expressions of GlyR and GlyT were evaluated in the keratinocytes after UV irradiation. These expressions were decreased after UV irradiation; however, they increased upon administration of FC or glycine. These increases were more prominent in the FC-treated group than in the glycine-treated group (Figure 1B, Figures S2A,B and S3).

### 2.2. FC Decreased the Expression of NOXs and Oxidative Stress in UV-Irradiated Keratinocytes

The expression levels of NOX1/2/4 were increased after UV irradiation and were decreased upon treatment with FC and glycine. This decrease was more prominent in the FC-treated group than in the glycine-treated group (Figure 1C and Figure S2C–E).

Oxidative stress was evaluated using the ratio of reduced glutathione (GSH) and oxidized glutathione (GSSG), SOD activity, and 8-hydroxy-2-deoxyguanosine (8-OHdG) levels. The ratio of GSH and GSSG has been frequently used to ascertain redox status [37,38].

The ratio of GSH and GSSG was decreased after UV irradiation and increased upon administration of FC or glycine. This increase in the ratio was more prominent in the FC-treated group than in the glycine-treated group (Figure 1D). The SOD activity was decreased after UV irradiation and increased upon administration of FC or glycine. The increase in SOD activity was more prominent in the FC-treated group than in the glycine-treated group (Figure 1E).

The increased level of 8-OHdG is one of most frequently used biomarkers for oxidative stress, as it is an oxidized metabolite [39,40]. The 8-OHdG levels were increased after UV irradiation and decreased upon treatment with FC and glycine. This decrease was more prominent in the FC-treated group than in the glycine-treated group (Figure 1F).

### 2.3. FC Treatment Decreased the Expression of p38 along with PKC, MITF, TRP1, TRP2, and TYR Activity in Melanocytes

**Figure 2:**
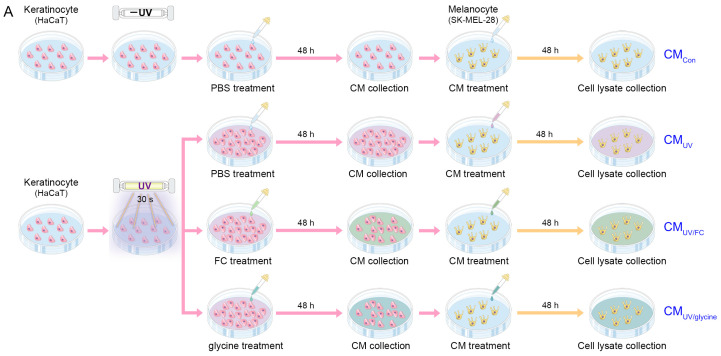

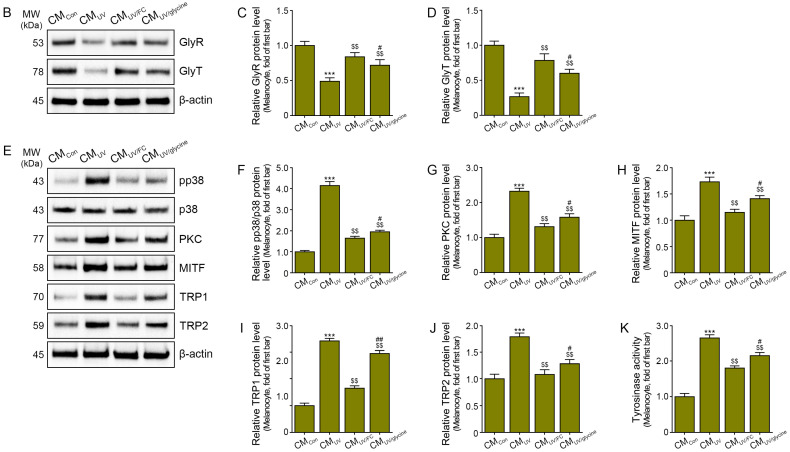
Regulation of p38, PKC, MITF, TRP1, TRP2, and TYR activity by FC and glycine in melanocytes. (**A**) Schematic diagram demonstrating melanocytes affected by conditioned media obtained from cultures of keratinocyte with UV-induced pigmentation for the evaluation of FC and glycine. (**B**) Protein expression of GlyR and GlyT in melanocytes treated with CM from UV-exposed keratinocytes with or without FC and glycine treatments. (**C**,**D**) Quantitative assessment of Western blot data presented in (**B**). (**E**) Protein expression of pp38, p38, PKC, MITF, TRP1, and TRP2 in melanocytes treated with CM from UV-exposed keratinocytes with or without FC and glycine treatments. (**F**–**J**) Quantitative assessment of Western blot data presented in (**E**). (**K**) Tyrosinase activity in melanocytes treated with CM from UV-exposed keratinocytes with or without FC and glycine treatments. Data are presented as the mean ± SD of three independent experiments. ***, *p* < 0.001, first bar vs. second bar; $$, *p* < 0.01, vs. second bar; #, *p* < 0.05; ##, *p* < 0.01, vs. third bar (Mann–Whitney U test). CM, conditioned media; FC, fermented fish collagen; GlyR, glycine receptor; GlyT, glycine transporter; MITF, microphthalmia-associated transcription factor; PBS, phosphate-buffered saline; PKC, protein kinase C; pp38, phosphorylated p38; SD, standard deviation; TRP1, tyrosinase-related protein-1; TRP2, tyrosinase-related protein-2; UV, ultraviolet.

UV irradiation affects keratinocytes, which in turn leads to increased melanogenesis in melanocytes. Thus, we designed an in vitro model by treating melanocytes with conditioned media (CM) used to culture keratinocytes. CM_Con_ referred to CM obtained from non-UV-irradiated keratinocytes that were treated with PBS. CM_UV_ referred to CM obtained from UV-irradiated keratinocytes that were treated with PBS. CM_UV/FC_ referred to CM obtained from UV-irradiated keratinocytes that were treated with FC. CM_UV/glycine_ referred to CM obtained from UV-irradiated keratinocytes that were treated with glycine. Thus, the melanocytes were treated using these CM (Figure 2A).

The expressions of GlyR and GlyT were increased after treatment with the CM_UV_ in the melanocytes. They were decreased after administration of the CM_UV/FC_ or CM_UV/glycine_. These decreases were more prominent in the CM_UV/FC_-treated cells than in the CM_UV/glycine_-treated cells (Figure 2B–D).

The expression ratio of phosphorylated p38 to p38 was increased after treatment with the CM_UV_ in the melanocytes. It was decreased after administration of the CM_UV/FC_ or CM_UV/glycine_. This decrease was more prominent in the CM_UV/FC_-treated cells than in the CM_UV/glycine_-treated cells (Figure 2E,F).

The expression of PKC was increased after treatment with the CM_UV_ in the melanocytes. It was decreased after administration of the CM_UV/FC_ or CM_UV/glycine_. This decrease was more prominent in the CM_UV/FC_-treated cells than in the CM_UV/glycine_-treated cells (Figure 2E,G).

The expression levels of MITF, TRP1, and TRP2 were increased after treatment with the CM_UV_ in the melanocytes. These expression levels were decreased upon administration of the CM_UV/FC_ or CM_UV/glycine_. These decreases were more prominent in the CM_UV/FC_-treated cells than in the CM_UV/glycine_-treated cells (Figure 2E,H–J).

TYR activity was increased after treatment with the CM_UV_ in the melanocytes. It was decreased upon administration of the CM_UV/FC_ or CM_UV/glycine_. This decrease was more prominent in the CM_UV/FC_-treated cells than in the CM_UV/glycine_-treated cells (Figure 2K).

### 2.4. FC Increased GlyR and GlyT and Decreased Oxidative Stress in the UV-Irradiated Animal Skin

**Figure 3:**
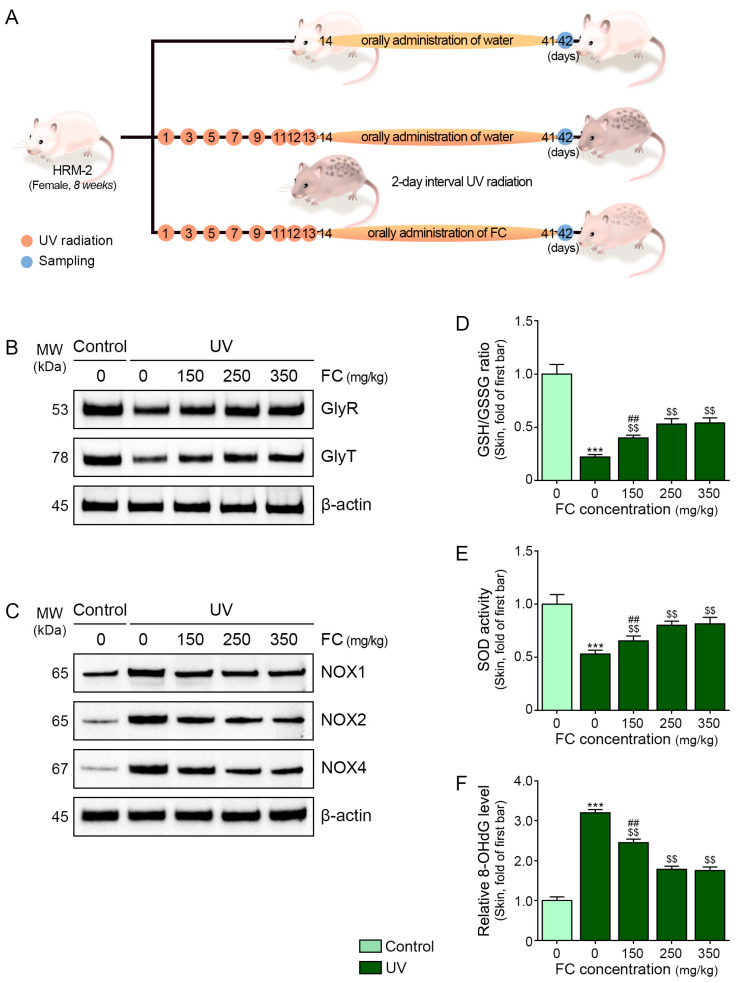
Regulation of GlyR and GlyT expression and oxidative stress by FC in UV-exposed animal skin. (**A**) Schematic diagram of the treatment of UV-exposed animal skin with FC. (**B**) Protein expression of GlyR and GlyT in UV-exposed animal skin following FC treatment was measured using Western blot. (**C**) Protein expression of NOXs in UV-exposed animal skin following FC treatment. (**D**) The increased level of GSH/GSSG ratio in UV-exposed animal skin following FC treatment. (**E**) The SOD activity in UV-exposed animal skin following FC treatment. (**F**) The increased level of 8-OHdG in UV-exposed animal skin following FC treatment. Data are presented as the mean ± SD of three independent experiments. ***, *p* < 0.001, first bar vs. second bar; $$, *p* < 0.01, vs. second bar; ##, *p* < 0.01, vs. fourth bar (Mann–Whitney U test). FC, fermented fish collagen; GlyR, glycine receptor; GlyT, glycine transporter; GSH, glutathione; GSSG, oxidized glutathione; NOX, nicotinamide adenine dinucleotide phosphate oxidase; SD, standard deviation; SOD, superoxide dismutase; UV, ultraviolet; 8-OHdG, 8-hydroxy-2-deoxyguanosine.

Since in vitro experiments revealed that FC treatment had a superior effect compared to glycine treatment on decreasing melanogenesis-related cell signal pathways, we evaluated only the effect of FC on melanogenesis, using different concentrations accompanied by UV irradiation in animal models (Figure 3A). Animal weight was measured weekly to evaluate whether FC intake affected the animal’s nutritional status and overall health status. The group that consumed the FC at doses of 150, 250, and 350 mg/kg showed no difference in weight gain compared to the group that consumed only water (Figure S4).

The expression levels of GlyR and GlyT were decreased after UV irradiation and were increased upon treatment with 150, 250, and 350 mg/kg of FC. This increase was not significantly different among the UV-irradiated animal skins treated with 250 and 350 mg/kg of FC (Figure 3B and Figure S5A,B).

The expression levels of NOX1/2/4 were increased after UV irradiation and were decreased upon treatment with 150, 250, and 350 mg/kg of FC. This decrease was not significantly different among the UV-irradiated animal skins treated with 250 and 350 mg/kg of FC (Figure 3C and Figure S5C–E).

The ratio of GSH and GSSG was decreased upon UV irradiation and was increased after treatment with 150, 250, and 350 mg/kg of FC. This increase was not significantly different among the UV-irradiated animal skins treated with 250 and 350 mg/kg of FC (Figure 3D).

The SOD activity was decreased upon UV irradiation and increased after treatment with 150, 250, and 350 mg/kg of FC. This increase was not significantly different among the UV-irradiated animal skins treated with 250 and 350 mg/kg of FC (Figure 3E).

The 8-OHdG level was increased upon UV irradiation and decreased after treatment with 150, 250, and 350 mg/kg of FC. This decrease was not significantly different among the UV-irradiated animal skins treated with 250 and 350 mg/kg of FC (Figure 3F).

### 2.5. FC Decreased Melanogenesis in UV-Irradiated Animal Skin

**Figure 4:**
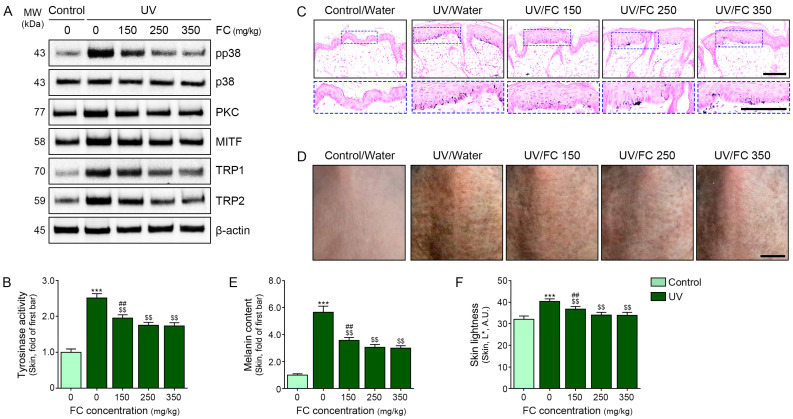
Regulation of melanogenesis upon treatment with different concentrations of FC in UV-exposed animal skin. (**A**) Protein expression of pp38, p38, PKC, MITF, TRP1, and TRP2 in UV-exposed animal skin following FC treatment. (**B**) Tyrosinase activity in UV-exposed animal skin following FC treatment. (**C**,**E**) Melanin content was determined using Fontana–Masson staining in UV-exposed animal skin with or without FC treatments. Scale bar = 100 µm. The blue dotted boxes are magnified images of the Fontana–Masson image. (**D**,**F**) Skin lightness in UV-irradiated animal skin with or without FC treatments. Scale bar = 500 µm. Data are presented as the mean ± SD of three independent experiments. ***, *p* < 0.001, first bar vs. second bar; $$, *p* < 0.01, vs. second bar; ##, *p* < 0.01, vs. fourth bar (Mann–Whitney U test). A.U., arbitrary unit; FC, fermented fish collagen; L*, lightness; MITF, microphthalmia-associated transcription factor; PKC, protein kinase C; pp38, phosphorylated p38; SD, standard deviation; TRP1, tyrosinase-related protein-1; TRP2, tyrosinase-related protein-2; UV, ultraviolet.

The expression levels of pp38/p38 and PKC were increased after UV irradiation and decreased after treatment with 150, 250, and 350 mg/kg of FC. This decrease was not significantly different among the UV-irradiated animal skins treated with 250 and 350 mg/kg of FC (Figure 4A and Figure S6A,B).

The expression levels of MITF, TRP1, and TRP2 were increased upon UV irradiation and decreased after treatment with 150, 250, and 350 mg/kg of FC. These decreases were not significantly different among the UV-irradiated animal skins treated with 250 and 350 mg/kg of FC (Figure 4A and Figure S6C–E).

The TYR activity was increased after UV irradiation and decreased after treatment with 150, 250, and 350 mg/kg of FC. This decrease was not significantly different among the UV-irradiated animal skins treated with 250 and 350 mg/kg of FC (Figure 4B).

Melanin content was evaluated using Fontana–Masson staining. It was increased after UV irradiation and decreased after treatment with 150, 250, and 350 mg/kg of FC. This decrease was not significantly different among the UV-irradiated animal skins treated with 250 and 350 mg/kg of FC (Figure 4C,E).

Skin lightness was evaluated using colorimetry. It was increased after UV irradiation and decreased after treatment with 150, 250, and 350 mg/kg of FC. This decrease was not significantly different among the UV-irradiated animal skins treated with 250 and 350 mg/kg of FC (Figure 4D,F).

## 3. Discussion

ROS is a major cause of increasing melanogenesis [41]. Thus, various antioxidants such as carotenoids and polyphenols have been evaluated as candidates for treatment of excessive-melanogenesis-related skin problems [41].

Collagen-derived protein sources such as gelatin and collagen peptide contain high amounts of proline and glycine [42,43]. Since these peptides are precursors for collagen synthesis, many studies have reported that collagen-derived proteins could increase collagen synthesis in the various connective tissues [44]. Oral collagen supplements are reported to increase human skin elasticity [45]. Collagen peptides increased collagen fiber and improved skin laxity in aged animals [46].

Since collagen-derived protein sources contain glycine, which is a known antioxidant, we speculated that FC—a fermented collagen that also contains glycine—could decrease melanogenesis.

Glycine showed an antioxidant effect via stimulating glutathione synthesis [47,48]. For the synthesis of GSH, the action of GlyT, which transports glycine into cytosol, is necessary [33]. Without the action of GlyT, glycine treatment would not be able to decrease oxidative stress [33].

GlyR is involved in glycine’s cytoprotective effect as it modulates the MAPK (JNK, ERK1/2, and p38) signaling pathways [49,50,51]. Glycine treatment decreased the expression of inflammatory cytokines such as TNF-α via GlyR [34,52]. Glycine treatment also decreased ROS via GlyR [36].

Fermented fish collagen (FC) is generated by fermentation of hydrolyzed fish collagen with *Lactobacillus plantarum* BJ21 and *Lactobacillus brevis* BJ20. By fermentation, the concentration of various amino acids is increased. In the case of glycine, the concentration increases to more than 12 times that of its concentration in non-fermented fish collagen.

FC contains glycine; thus, we evaluated the effect of treatment with FC on melanogenesis and evaluated whether a decrease in oxidative stress was involved therein. First, we evaluated whether FC affected the expression levels of GlyR and GlyT in UV-irradiated keratinocytes. The expressions of both GlyT and GlyR were decreased after UV irradiation and increased after treatment with FC. Along with this, oxidative stress, which was evaluated using GSH/GSSG, SOD activity, and 8-OHdG expression, was increased after UV irradiation and decreased upon treatment with FC in the keratinocytes. Next, we evaluated whether FC-treatment-induced changes in keratinocytes affected melanocytes and decreased melanogenesis. When CM from UV-irradiated keratinocytes was used to treat the melanocytes, p38 and PKC expression levels were increased. These expressions were decreased after treatment with CM from FC-treated keratinocytes. Moreover, key melanogenesis signaling pathways such as MITF/TRP-1/TRP-2 were increased upon treatment with the CM obtained from UV-irradiated keratinocytes. However, these were decreased after treatment with the CM obtained from FC-treated keratinocytes. These changes were more prominently observed in the groups treated with FC than in those treated with glycine. These results suggest that FC could decrease melanogenesis-related signaling pathways in melanocytes, likely through keratinocyte modulation.

In the in vivo investigation, FC, which was orally administered to mice, increased the expression of GlyR and GlyT in the skin, similar to our observations in the in vitro study. In vivo, GSH/GSSG and SOD activity were decreased upon UV irradiation and increased after FC treatment; expression levels of NOX1/2/4 and 8-OHdG were increased after UV irradiation and decreased after treatment with FC. Similarly, the activity of the melanogenesis signaling pathways p38, PKC, MITF, TRP1, TRP2, and TYR was increased upon UV irradiation and decreased upon treatment with FC. These effects of FC on melanogenesis were concentration dependent. The effect of 150 mg/kg of FC was lower than that shown by 250 or 350 mg/kg of FC.

Glycine has been reported to exert various cytoprotective effects [36,49,50,51]. Since FC contains glycine, it would be reasonable to say that FC treatment decreased oxidative stress via GlyR. In our study, the FC treatment increased the expression levels of both GlyR and GlyT and decreased oxidative stress. The exact mechanism through which FC treatment increased expression of GlyR and GlyT should be evaluated in future studies. Moreover, the serum level or tissue level of the glycine or the FC were not evaluated in this study. Thus, it cannot be concluded that ingested FC reached the tissue and thus the tissue-reached FC directly increased the GlyT or GlyR. To evaluate the exact mechanisms by which FC increases GlyT or GlyR, the tissue and serum levels of the FC and glycine should be evaluated in a future study. Even though the exact mechanism via which the FC decreased oxidative stress was not fully evaluated in this study, both the in vitro and in vivo studies showed that the FC decreased oxidative stress after UV radiation, which is possibly associated with decreasing melanogenesis.

Facial hyperpigmentation, such as melasma and post-inflammatory hyperpigmentation, is a stressful condition that causes various aesthetic problems [53,54]. Numerous studies aimed at finding suitable treatments for hyperpigmentation have been conducted. As topical treatment, skin lightening agents such as retinoids, hydroquinone (HQ), 5% cysteamine cream, and tranexamic acid have been used [55,56,57]. However, these agents could cause complications. Side effects of HQ such as erythema, burning sensation, pruritus, and desquamation have been reported [58,59,60,61]. Moreover, HQ induces paradoxical permanent hyperpigmentation [53]. Use of retinoids may also lead to complications such as dryness, burning, dermatitis, and post-inflammatory hyperpigmentation [62,63]. Topical tranexamic acid had side effects including transient erythema, scaling, and dryness [64]. Moreover, tranexamic acid is not free from a theoretical risk of causing a thrombotic event [64]. Cysteamine cream could cause transient dryness and burning [65,66]. Its malodorous nature is also a limitation for use [65,66].

Collagen supplements have been used without serious complications [67]. Collagen from animals could increase the risk of disease transmission [67]. Collagen supplements also cause allergic reactions [67]. Even though we did not evaluate the long-term complications of FC in this study, it is expected that FC may decrease the risk of local skin complications such as dryness or erythema. FC could be a good candidate for treating hyperpigmentation with considerably fewer complications than other current treatments. In future studies, the long-term effects of FC should be evaluated.

## 4. Materials and Methods

### 4.1. FC Preparation and Analysis

#### 4.1.1. FC Preparation

FC (GABALAGEN) was acquired from Marine Bioprocess Co., Ltd. (Busan, Republic of Korea). Before fermentation, fish collagen from Geltech Co., Ltd. (Busan, Republic of Korea) is hydrolyzed at 55 °C ± 2 °C for 12 h with prozyme (Bisionbiochem Co., Ltd, Seoul, Republic of Korea). The FC production required two consecutive fermentations by *Lactobacillus brevis* BJ20 (accession No. KCTC 11377BP) and *Lactobacillus plantarum* BJ21 (accession No. KCTC 18911P). A seed medium composed of 3% yeast extract (Choheung, Ansan, Korea), 1% glucose (Choheung, Ansan, Korea), L-glutamic acid 1% (Samin chemical, Siheung, Korea), and 95% water was sterilized for 15 min at 121 °C before being inoculated with 0.002% BJ20 and 0.002% *Lactobacillus Plantarum* BJ21. These microorganisms were then cultured for 24 h at 37 °C separately. For the first fermentation, 10% (*v*/*v*) of the *Lactobacillus brevis* BJ20 cultured seed medium was fermented in a fermentation medium (yeast extract 2%, glucose 0.28%, hydrolyzed fish collagen 29% (Geltech Co., Ltd., Busan, Republic of Korea), L-glutamic acid 5.5% (Samin chemical, Siheung, Korea), water 63.22%) at 37 °C for 24 h. Then, 10% (*v*/*v*) of BJ21 cultured seed medium was added and fermented at 37 °C for another 24 h. The fermentation medium was sterilized and spray-dried to prepare FC powder samples.

#### 4.1.2. Amino Acid Analysis

The FC was hydrolyzed and analyzed using an amino acid analyzer (L-8900, Hitachi, Tokyo, Japan).

Briefly, 10 mg of the FC was hydrolyzed with 10 mL of 6.0 N HCl in a sealed-vacuum ampoule at 110 °C for 24 h for amino acid composition analysis. The HCl was removed from the hydrolyzed sample on a rotary evaporator and brought to a known volume (10 mL). Then the amino acids were determined using an L-8900 amino acid analyzer (Hitachi, Tokyo, Japan). For determination of free amino acids, 3.0 g of the FC and an equal volume of 16% trichloroacetate solution were homogenized using a vortex mixer for 2 min. The homogenized sample was centrifuged at 3000 rpm for 15 min. The supernatants from the first and second extraction were combined and filtered through a Whatman No. 41 filter paper. The supernatant was acidified to pH 2.2 with a 10 M HCl solution and then diluted to 50 mL with distilled water. Samples were then analyzed using the same amino acid analyzer.

It was confirmed that the FC thus produced contained 1% glycine among the free amino acids (Table S1).

#### 4.1.3. High-Performance Liquid Chromatography (HPLC) Analysis

A 0.1 g standard sample was dissolved in 100 mL of distilled water (DW) in a volumetric flask to prepare a standard solution, filtered through a polytetrafluoroethylene syringe filter (25 mm/0.2 μm), and stored at −80 °C. For the 5% aqueous sample solution, 5 g of FC was dissolved in DW in a 100 mL volumetric flask and filtered using a polytetrafluoroethylene syringe filter.

In our study, a Dionex U3000 series HPLC system (Thermo Fisher, Waltham, MA, USA) equipped with a UV detector was utilized, and the flow rate was lowered to 1 mL/min. The samples were analyzed using UV–Vis spectrophotometry at a wavelength of 338 nm. The amount of GABA in the RG was calculated using the following equation:

Substance (mg/g) = Measurement (mg/mL) × Dilution factor ÷ Amount (g) × 100 (mL)

It was confirmed that the FC thus produced contained 1% glycine.

### 4.2. In Vitro Model

Human keratinocytes (HaCaT) were distributed and used by Professor Jeong Hee Hong’s team at Gachon University. The keratinocytes were cultured in Dulbecco’s modified Eagle’s medium (DMEM; HyClone, Logan, UT, USA) at 37 °C with 5% CO_2_. Human melanocytes (SK-MEL-28) were purchased from American Type Culture Collection (ATCC; Manassas, VA, USA) and cultured in Minimum Essential Medium (MEM; cytiva, Washington, DC, USA) at 37 °C with 5% CO_2_.

In vitro experiments were conducted in four stages to investigate the impact of the FC and glycine on the keratinocytes and melanocytes. Initially, the cytotoxicity of the FC was assessed by culturing keratinocytes to a confluence of over 90%, followed by incubation in phosphate-buffered saline (PBS) or varying concentrations (1–100,000 μg/mL) of FC for the CCK-8 assay (Figure S1A). Next, to determine the optimal FC concentrations, keratinocytes were exposed to UV radiation (UV lamp with 306 nm peak wavelength) for 30 sec and subsequently treated with PBS or 50, 100, 250, 500, or 1000 μg/mL of FC for 48 h, with cell lysates collected for SOD activity analysis (Figure S1C). After confirming 250 μg/mL as the optimal FC concentration, keratinocytes were exposed to UV radiation and then treated for 48 h with PBS, 250 μg/mL of FC, or 2.5 μg/mL of glycine. Control groups, not exposed to UV radiation, were similarly incubated with PBS. Following the 48 h incubation, cell lysates were collected for protein analysis, and supernatant was collected for treatment with melanocytes. In the fourth stage, a melanocyte model was used treated with CM of keratinocyte for 48 h (Figure 2A). Cell lysates from all the melanocyte groups were then collected for subsequent protein analysis.

### 4.3. In Vivo Model

#### 4.3.1. Mouse Model and Maintenance

Female hairless HRM-2 (6-week-old) mice were obtained from the Central Laboratory Animal Center (Incheon, Republic of Korea) and stabilized in our facility for 2 weeks before the experiments. All the animals were housed under conditions of constant temperature 20–24 °C and humidity 45–55% and were allowed ad libitum access to food and water. This study was conducted with approval from the Gachon University Animal Experiment Ethics Committee (IACUC, approval number LCDI-2022-0107).

#### 4.3.2. Experimental Design

The stabilized animals were randomly assigned to five groups. Four of these groups underwent UV radiation exposure according to methods previously outlined [68]. In summary, a UV lamp (Sankyo) exposing a 306 nm peak wavelength was used to administer 200 mJ/cm^2^ of UV radiation to the mice’s backs once every 2 days for 10 days, followed by daily exposure for the subsequent 3 days. Subsequently, they received oral administrations of water or FC at doses of 150, 250, or 350 mg/kg (5 mL/kg body weight) and were exposed to UV light once every 2 days for a period of 28 days [69]. The FC was mixed with drinking water and did not smell or taste strongly enough to make it difficult to ingest. After this treatment period, the animals’ skin was harvested (Figure 3A).

#### 4.3.3. Skin Lightness

Skin color was assessed using a CR-10 color reader (Konica Minolta Sensing, Inc., Sakai, Osaka, Japan), with L* (lightness) measured in the CIELAB color space (International Commission on Lighting, Vienna, Austria). Measurements were averaged over a cycle of 10 readings taken on the 42nd day following the initial UV irradiation, corresponding to 28 days after the commencement of treatment.

### 4.4. Sample Preparation

#### 4.4.1. Protein Isolation

Protein extraction followed the protocol outlined in the EzRIPA Lysis kit (ATTO Corporation, Tokyo, Japan). Cells were initially washed with PBS and then scraped into 1 mL of RIPA buffer. For skin samples, 50 mg of tissue was cut into small pieces, diluted in 0.6 mL of RIPA buffer, and homogenized by sonication for 10 cycles with 40 s on and 60 s off, followed by incubation on ice for 10 min to enhance protein solubilization. Both cell and tissue samples underwent further sonication (high power, 10 s on and 60 s off) and were subsequently centrifuged at 14,000× *g* for 15 min at 4 °C to isolate proteins. Protein concentrations were determined using a bicinchoninic acid assay kit (BCA kit, Thermo Fisher Scientific).

#### 4.4.2. Paraffin-Embedded Skin Tissue Block

Skin tissues were fixed in cold 4% paraformaldehyde (Sigma-Aldrich, St. Louis, MO, USA) for 48 h then transferred to cassettes and rinsed with distilled water (DW). The samples were then subjected to a series of dehydration steps in a tissue processor (Leica, Wetzlar, Germany), sequentially immersed in 95% and 99% ethanol (Duksan), then in xylene (Duksan), and finally embedded in paraffin (Leica). The paraffin-embedded tissue blocks were processed into paraffin blocks using an embedder, sectioned at 7 μm thickness using a microtome (Leica), placed on pre-coated slides, incubated overnight at 60 °C, and then attached to the slides.

### 4.5. Cell Viability

To evaluate the cytotoxicity of the FC, keratinocytes were seeded in 96-well plates at a density of 1 × 10^4^ cells per well. Once the wells reached full confluence, the cells were exposed to FC at concentrations ranging from 1 to 100,000 μg/mL for 24 h. After treatment, the medium was aspirated, and the cells were gently washed with DPBS (Gibco). Then, 10 μL of CCK-8 reagent (Sigma-Aldrich) was added to each well along with 90 μL of growth medium, and the plate was incubated at 37 °C for 2 h. The optical density of each well was measured at 450 nm using a microplate reader. Each experiment was performed in triplicate to ensure accuracy and reproducibility of the results.

### 4.6. GSH/GSSG Ratio, SOD Activity, and Tyrosinase Activity

GSH/GSSG ratio (cat. no. V6611, Promega, Madison, WI, USA), NADPH/NADP+ ratio (cat. no. ab65349, Abcam, Waltham, MA, USA), SOD activity (cat. no. ab65354, Abcam), and tyrosinase activity (cat. no. ab252899, Abcam) in the keratinocytes, the melanocytes treated with CM from keratinocytes, and the animal skin were measured using appropriate kits according to the manufacturer’s instructions.

### 4.7. Western Blot

Thirty micrograms of cell lysate or skin protein was mixed with 4× LDS sample buffer (Thermo Fisher Scientific) and 10× sample reducing agent (Thermo Fisher Scientific). The protein mixture was heated at 70 °C for 10 min to denature the proteins, which were then separated by 10% sodium dodecyl sulfate-polyacrylamide gel electrophoresis (SDS-PAGE) using MOPS buffer (Invitrogen, Waltham, MA, USA) at 200 V for 25 min. The separated proteins were transferred to PVDF membranes (Millipore, Burlington, MA, USA) using a semidry transfer system at 1 A current for 10 min.

To minimize nonspecific binding, the PVDF membranes were incubated in 0.1% Tween 20 (SPL, Pocheon, Republic of Korea) and 5% skim milk (LPS Solution, Daejeon, Republic of Korea) in Tris-buffered saline (TTBS) for 1 h at room temperature. After washing three times with 0.1% TTBS, the membranes were incubated with appropriately diluted primary antibodies overnight at 4 °C (Table S2). After three more washes with 0.1% TTBS, the membranes were incubated with horseradish peroxidase-conjugated secondary antibody (1:10,000, Vector Laboratories, Newark, CA, USA) for 1 h at room temperature.

Protein bands were visualized using chemiluminescence solution and detected using the ChemiDoc Imaging System (Bio-Rad, Hercules, CA, USA). For quantitative analysis of the protein bands, the intensity was measured using ImageJ software version 1.53s (NIH). The beta-actin band served as a loading control to ensure equal loading of samples. Each experimental group was compared to control samples to assess relative changes in protein expression levels.

### 4.8. Enzyme-Linked Immunosorbent Assay (ELISA)

Microplates were first incubated overnight at 4 °C using a mixed buffer of 100 nM carbonate and bicarbonate (pH 9.6) and washed three times with 0.1% Tween 20 in phosphate-buffered saline (TPBS) to remove unbound material. To minimize nonspecific protein binding, the microplates were incubated overnight at 4 °C with 5% skim milk in 0.1% TPBS (LPS solution). After three additional washes with 0.1% TPBS, 30 μg of cell lysated or tissue protein sample was added to each well and incubated overnight at 4 °C. After one more wash with 0.1% TPBS, the wells were incubated overnight at 4 °C with primary antibodies diluted in PBS (Table S2). After a final wash with PBS, horseradish peroxidase-conjugated secondary antibody (1:1000, Vector Laboratories) was added and incubated at room temperature for 4 h.

To detect protein expression, tetramethylbenzidine (TMB) solution (Sigma-Aldrich) was added to each well and incubated for 15–20 min at room temperature. The reaction was stopped by adding sulfuric acid (Sigma-Aldrich), and the absorbance was measured at 450 nm using a microplate reader. The absorbance OD measurements from the same amount of protein were normalized to the value of the control group (first bar). Each analysis was performed in triplicate to ensure reliability and consistency of the results.

### 4.9. Fontana–Masson Staining

Fontana–Masson staining was performed according to the manufacturer’s protocol (Scytek, Logan, UT, USA). Initially, skin tissue sections were deparaffinized and rehydrated by sequential immersion in xylene and graded ethanol solutions (100–70%). The sections were then incubated in Fontana ammoniacal silver solution at 60 °C for 30 min. After rinsing three times with DW, the slides were treated with 0.2% gold chloride solution and then with 5% sodium thiosulfate solution. To visualize the nuclei, the sections were stained with Nuclear Fast Red solution. After staining, the sections were dehydrated and mounted using DPX mounting solution (Sigma-Aldrich). The stained tissue sections were then scanned using a slide scanner (Motic Scan Infinity 100) and images were captured randomly.

Quantitative analysis of melanin content was performed using ImageJ software version 1.53s (NIH), the intensity of black staining being considered positive melanin staining. The software extracted and quantified the amount of black in the image. To assess relative changes in melanin levels, each experimental group was compared to control samples.

### 4.10. Statistical Analysis

The Kruskal–Wallis test was performed for group comparisons, followed by the Mann–Whitney U test for post hoc comparisons. The results were expressed as mean ± SD. All statistical analyzes were performed using SPSS version 26 (IBM, Armonk, NY, USA). Statistical significance is indicated in the legend of each figure.

## 5. Conclusions

In conclusion, FC treatment increased the expression of both of GlyR and GlyT in UV-irradiated keratinocytes. FC treatment also decreased NOX1/2/4 expression and oxidative stress in UV-irradiated keratinocytes. Keratinocytes treated using FC also affected melanocytes and led to a decrease in the activity of the melanogenesis signal pathways p38, PKC, MITF, TRP1, and TRP2, and TYR. FC treatment also decreased the expression of GlyR and GlyT in the skin of UV-irradiated mice, which was accompanied by a decrease in the expression of melanogenesis signaling pathways and melanin content. FC could thus be a candidate for treatment of hyperpigmentation-related skin problems.

## Data Availability

All data are contained within this article.

## Supplementary Materials

The following supporting information can be downloaded at https://www.mdpi.com/article/10.3390/md22090421/s1. Figure S1: In vitro study for optimization of FC concentrations. Figure S2: Regulation of GlyR, GlyT, and NOXs expression by FC and glycine in UV-exposed keratinocytes. Figure S3**:** Regulation of GlyR and GlyT expression by FC and glycine in UV-exposed keratinocytes with different antibodies. Figure S4: Body weight changes according to FC administration. Figure S5: Regulation of GlyR, GlyT, and NOXs expression by different concentrations of FC in UV-exposed animal skin. Figure S6: Regulation of p38, PKC, MITF, TRP1, and TRP2 expression by different concentrations of FC in UV-exposed animal skin. Table S1: Free amino acid analysis of FC. Table S2: List of antibodies for Western blot (WB) and Enzyme-linked immunosorbent assay (ELISA).
