# Supplementary material for: Fermented Fish Collagen Attenuates Melanogenesis via Decreasing UV-Induced Oxidative Stress
Source: Mar Drugs. 2024 Sep 15;22(9):421. doi: 10.3390/md22090421 (PMC11433465; doi:10.3390/md22090421)
Supplement: Supplementary file 1 [file marinedrugs-22-00421-s001.zip › marinedrugs-3117492-supplementary.pdf]

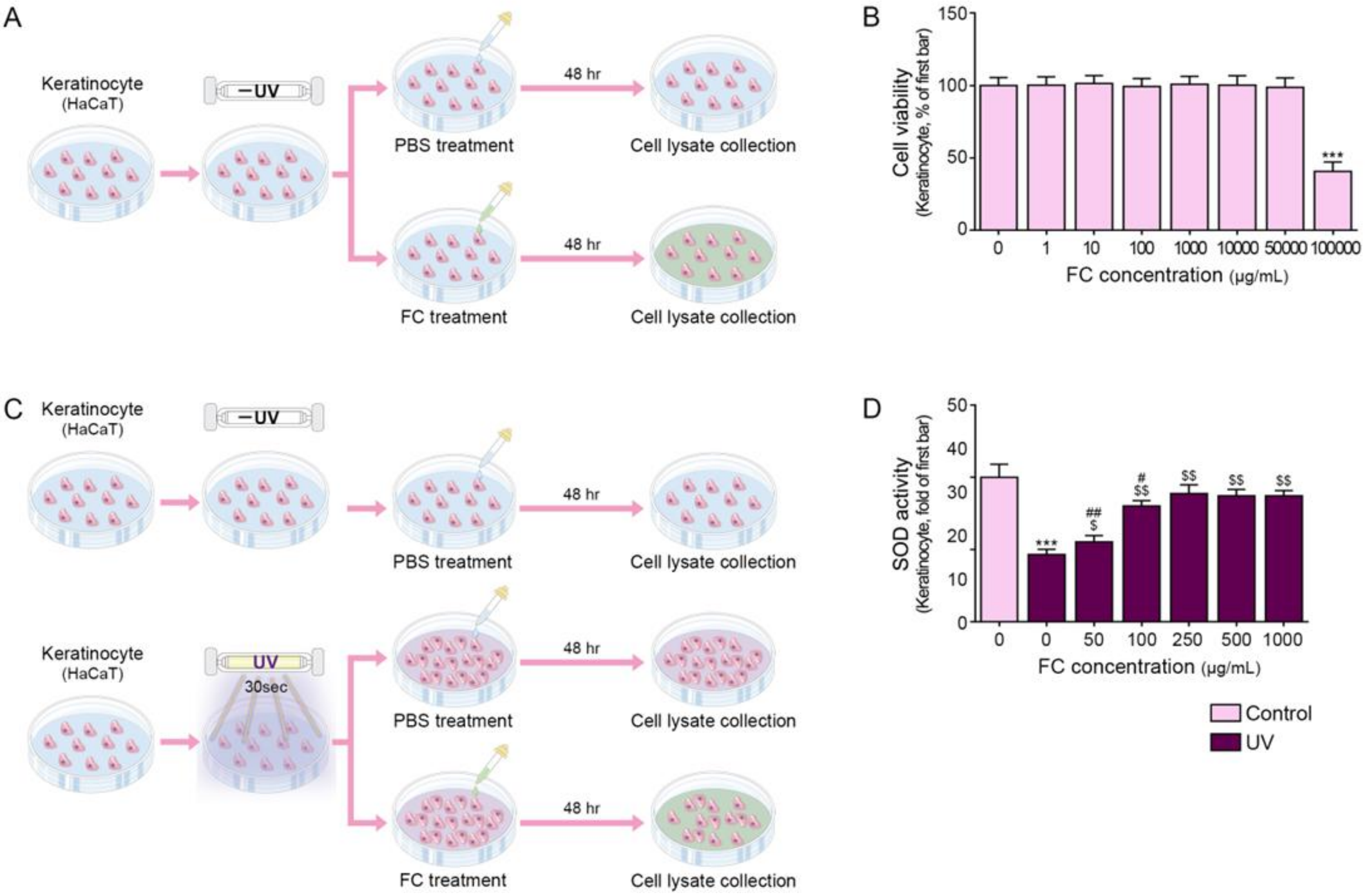

Figure S1. *In vitro* study for optimization of FC concentrations. (**A**) Schematic diagram demonstrating the treatment of keratinocytes with FC for cytotoxic effects. (**B**) The cell viability of keratinocytes following FC treatment was measured using CCK-8 assay. (**C**) Schematic diagram demonstrating the treatment of keratinocytes with FC for suitable concentration. (**D**) The SOD activity in UV-exposed keratinocytes following FC treatment was measured. Data are presented as the mean ± SD of three independent experiments. ***, $p < 0.001$, vs first bar; $, $p < 0.05$, $$, $p < 0.01$, vs second bar; #, $p < 0.05$, ##, $p < 0.01$, vs fifth bar (Mann–Whitney U test). FC, fermented fish collagen; PBS, phosphate-buffered saline; SD, standard deviation; SOD, superoxide dismutase; UV, ultraviolet.

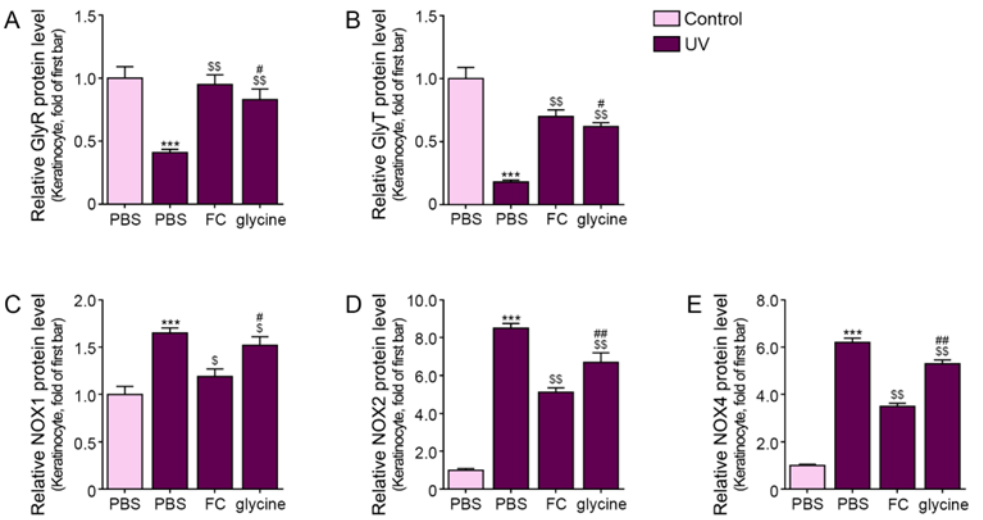

Figure S2. Regulation of GlyR, GlyT, and NOXs expression by FC and glycine in UV-exposed keratinocytes. (**A**,**B**) Quantitative assessment of western blot data presented in Figure 1B. (**C**–**E**) Quantitative assessment of western blot data presented in Figure 1C. Data are presented as the mean

± SD of three independent experiments. ***, $p < 0.001$, first bar vs second bar; $, $p < 0.05$, $$, $p < 0.01$, vs second bar; #, $p < 0.05$, ##, $p < 0.01$, vs third bar (Mann–Whitney U test). FC, fermented fish collagen; GlyR, glycine receptor GlyT, glycine transporter; NOX, nicotinamide adenine dinucleotide phosphate oxidase; PBS, phosphate-buffered saline; SD, standard deviation; UV, ultraviolet.

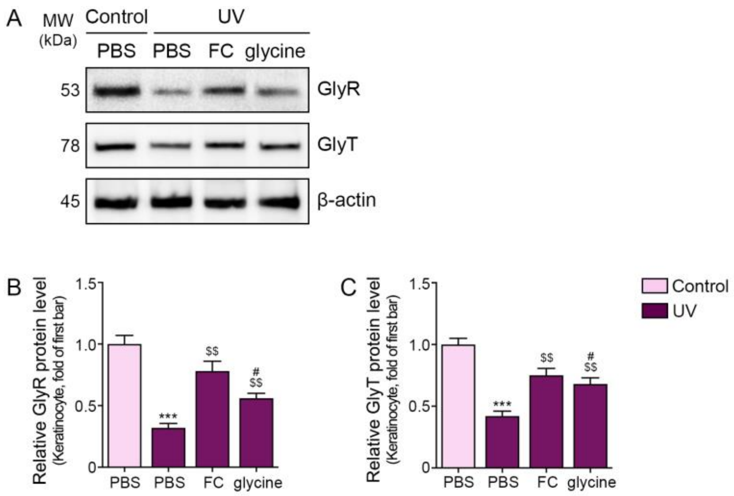

Figure S3. Regulation of GlyR and GlyT expression by FC and glycine in UV-exposed keratinocytes with different antibodies. (**A**) Protein expression of GlyR and GlyT in UV-exposed keratinocytes following FC and glycine treatment. (**B**,**C**) Quantitative assessment of western blot data. Data are presented as the mean ± SD of three independent experiments. ***, $p < 0.001$, first bar vs second bar; $$, $p < 0.01$, vs second bar; #, $p < 0.05$, vs third bar (Mann–Whitney U test). FC, fermented fish collagen; GlyR, glycine receptor GlyT, glycine transporter; PBS, phosphate-buffered saline; SD, standard deviation; UV, ultraviolet.

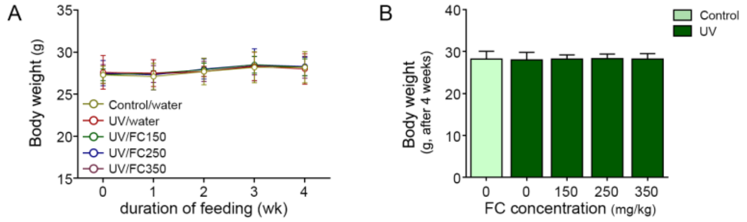

Figure S4. Body weight changes according to FC administration. (**A**) Body weight changes by week during the substance administration period. (**B**) Body weight changes after 4 weeks of substance administration. Data are presented as the mean ± SD of three independent experiments. FC, fermented fish collagen; SD, standard deviation.

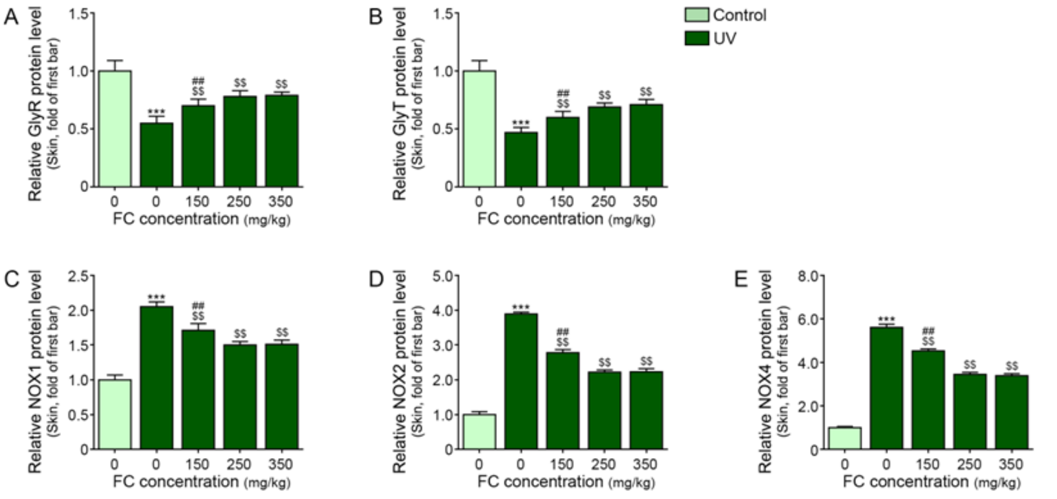

Figure S5. Regulation of GlyR, GlyT, and NOXs expression by different concentrations of FC in UV-exposed animal skin. (**A**,**B**) Quantitative assessment of western blot data presented in Figure 3B. (**C**–**E**) Quantitative assessment of western blot data presented in Figure 3C. Data are presented as the mean ± SD of three independent experiments. ***, $p < 0.001$, first bar vs second bar; $$, $p < 0.01$, vs second bar; ##, $p < 0.01$, vs fourth bar (Mann–Whitney U test). FC, fermented fish collagen; GlyR, glycine receptor GlyT, glycine transporter; NOX, nicotinamide adenine dinucleotide phosphate oxidase; SD, standard deviation.

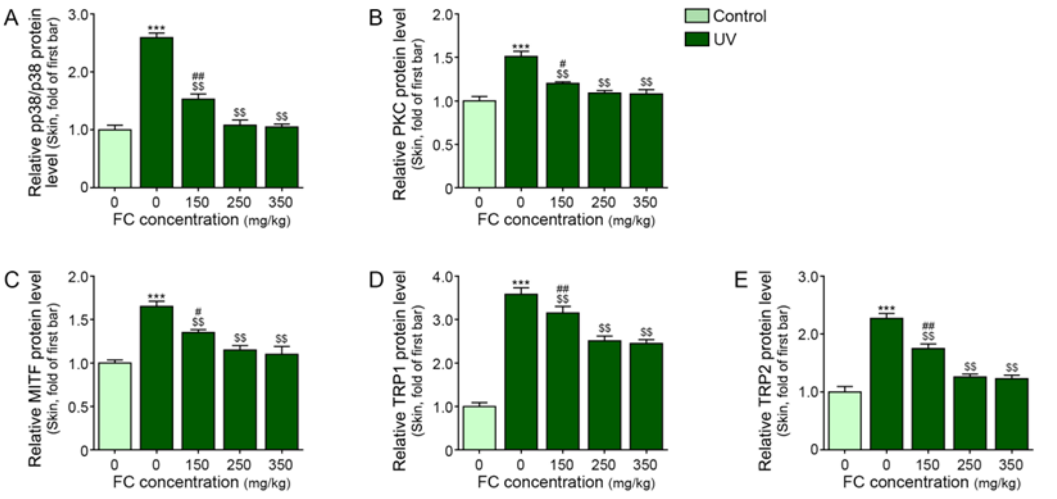

Figure S6. Regulation of p38, PKC, MITF, TRP1, and TRP2 expression by different concentrations of FC in UV-exposed animal skin. (**A**–**E**) Quantitative assessment of western blot data presented in Figure 4A. Data are presented as the mean ± SD of three independent experiments. ***, $p < 0.001$, first bar vs second bar; $$, $p < 0.01$, vs second bar; #, $p < 0.05$, ##, $p < 0.01$, vs fourth bar (Mann–Whitney U test). FC, fermented fish collagen; MITF, microphthalmia-associated transcription factor; PKC, protein kinase C; pp38, phosphorylated p38; SD, standard deviation; TRP1, tyrosinase-related protein-1; TRP2, tyrosinase-related protein-2; UV, ultraviolet.

Table S1. Free amino acid analysis of FC.

Fish collagen		Fermented fish collagen (FC)	
Amino acid	mg/g	Amino acid	mg/g
Glycine	0.9	Glycine	12.3
Alanine	1.5	Alanine	23.3
Lysine	0.0	Lysine	5.3
Methionine	1.2	Methionine	0.2
Arginine	1.3	Arginine	15.7
Isoleucine	3.1	Isoleucine	7.0
Phenylalanine	1.1	Phenylalanine	6.3
Histidine	0.0	Histidine	0.4
Tyrosine	0.3	Tyrosine	3.7
Serine	0.1	Serine	3.4
Glutamic acid	0.0	Glutamic acid	29.9
Leucine	0.3	Leucine	7.2
Valine	1.8	Valine	9.4
Aspartic acid	0.2	Aspartic acid	2.2
Threonine	0.1	Threonine	6.5
Proline	0.0	Proline	0.0
Hydroxy proline	0.1	Hydroxy proline	0.4
γ-aminobutyric acid	0.6	γ-aminobutyric acid	85.7
Cystine	0.8	Cystine	1.8

Table S2. List of antibodies for Western blot (WB) and Enzyme-linked immunosorbent assay (ELISA).

Antibody	Company	Catalog No.	Dilution rate	
			WB	ELISA
GlyR	biorbyt	orb523605	1:1,000	-
	biorbyt	orb522515	1:1,000	
GlyT	biorbyt	orb157205	1:1,000	-
	biorbyt	orb541708	1:1,000	
8-OHdG	GeneTex	GTX41980	-	1:500
NOX1	Affinity	DF8684	1:1,000	
NOX2	Affinity	DF86520	1:1,000	
NOX4	Affinity	DF6924	1:1,000	
p38	Cell Signaling	9212	1:1,000	-
pp38	Cell Signaling	4511	1:1,000	-
PKC	Bioss	BS-3531R	1:500	-
MITF	LSBIO	LS-C117668	1:500	-
TRP1	Santa Cruz	sc-392736	1:500	-
TRP2	Invitrogen	PA5-79481	1:500	-
β-actin	Cell signaling	4967	1:1,000	-
